# A case of intraductal tubulopapillary neoplasm of the pancreas in a branch duct: a rare case report and literature review

**DOI:** 10.1186/s12876-021-01744-2

**Published:** 2021-04-13

**Authors:** Atsushi Yamaguchi, Takuro Hamada, Kaoru Wada, Riho Moriuchi, Kanae Tao, Hirona Konishi, Yuzuru Tamaru, Ryusaku Kusunoki, Toshio Kuwai, Hirotaka Kouno, Kohei Ishiyama, Naoto Hadano, Takeshi Sudo, Naoyuki Toyota, Junichi Zaitsu, Kazuya Kuraoka, Hiroshi Kohno

**Affiliations:** 1 Department of Gastroenterology, National Hospital Organization Kure Medical Center and Chugoku Cancer Center, Kure, Hiroshima Prefecture Japan; 2 Department of Surgery, National Hospital Organization Kure Medical Center and Chugoku Cancer Center, Kure, Hiroshima Prefecture Japan; 3 Department of Radiology, National Hospital Organization Kure Medical Center and Chugoku Cancer Center, Kure, Hiroshima Prefecture Japan; 4 Department of Pathology, National Hospital Organization Kure Medical Center and Chugoku Cancer Center, Kure, Hiroshima Prefecture Japan

**Keywords:** Intraductal tubulopapillary neoplasm (ITPN), Branch of pancreatic duct, Main pancreatic duct, Case report, Pancreatic cancer

## Abstract

**Background:**

Intraductal tubulopapillary neoplasm (ITPN) of the pancreas is a new disease concept defined by the World Health Organization in 2010. ITPN progresses with tubulopapillary growth in the pancreatic duct and is known to have a fair prognosis. Localization in the main pancreatic duct (MPD) is one characteristic. There are few case reports of ITPN in a branch of the pancreatic duct (BD).

**Case presentation:**

We encountered a case of ITPN localized in BD. An 85-year-old man was followed after colonic surgery for rectal carcinoma. An abdominal computed tomography scan revealed a cystic mass in the pancreatic head and further examination was done. A T2 weighted intension picture in magnetic resonance imaging showed a 20 mm cystic lesion with an internal mass of 15 mm. Duodenal papilla were slightly open and endoscopic retrograde pancreatography revealed mild and diffuse dilatation of the main pancreatic duct and mucin in the MPD. In consideration with the image examinations, we diagnosed the tumor as an intraductal papillary mucinous neoplasm with carcinoma because of its large mural nodule (> 10 mm in size) in a cyst. Consequently, a pancreaticoduodenectomy was performed. Macroscopically, a white solid tumor sized 2.5 × 1.8 × 1.0 was identified in the head of the pancreas. The cut surface of the resected pancreas showed a side-branch type intraductal tumor with tubulopapillary architecture without mucin secretion. Immunohistochemical staining was positive for MUC1, and negative for MUC2 and MUC5AC. The final diagnosis was determined to be pancreatic ITPN from BD. At the time of this report (48 months post-surgery), the patient remains disease-free without evidence of recurrence.

**Conclusion:**

ITPNs localized in BD are rare and diagnosis prior to surgery is difficult. In our case, the shape was round, not papillary, and with little fluid. These characteristics are different from a branch duct type IPMN and can be a clue to suspect ITPN in BD.

## Background

Intraductal tubulopapillary neoplasm (ITPN) of the pancreas is a new disease concept defined by the World Health Organization in 2010 [1]. ITPN progresses with tubulopapillary growth in the pancreatic duct and is known to have relatively better prognosis than pancreatic ductal adenocarcinoma. Intraductal pancreatic tumors are divided into ITPN, intraductal papillary mucinous neoplasm (IPMN), and pancreatic intraepitheral neoplasia. The frequency of ITPN is rare and only 3% among the three tumors and 0.9% of all pancreatic exocrine tumors [2], thus its clinical and pathological features have not been fully understood. Many ITPNs have grown in the main pancreatic duct (MPD) and are accompanied by dilatation of upstream MPD and mimic pancreatic ductal adenocarcinoma (PDAC)[3]. ITPN growing only in a branch duct (BD) is rare (5%, 2/41) [3] and there are few case reports [3–6]. Accordingly, it is difficult to recognize the difference between ITPN in MPD and in BD. We experienced a case of ITPN localized in BD and report this case along with a literature review.

## Case presentation

**Fig. 1:**
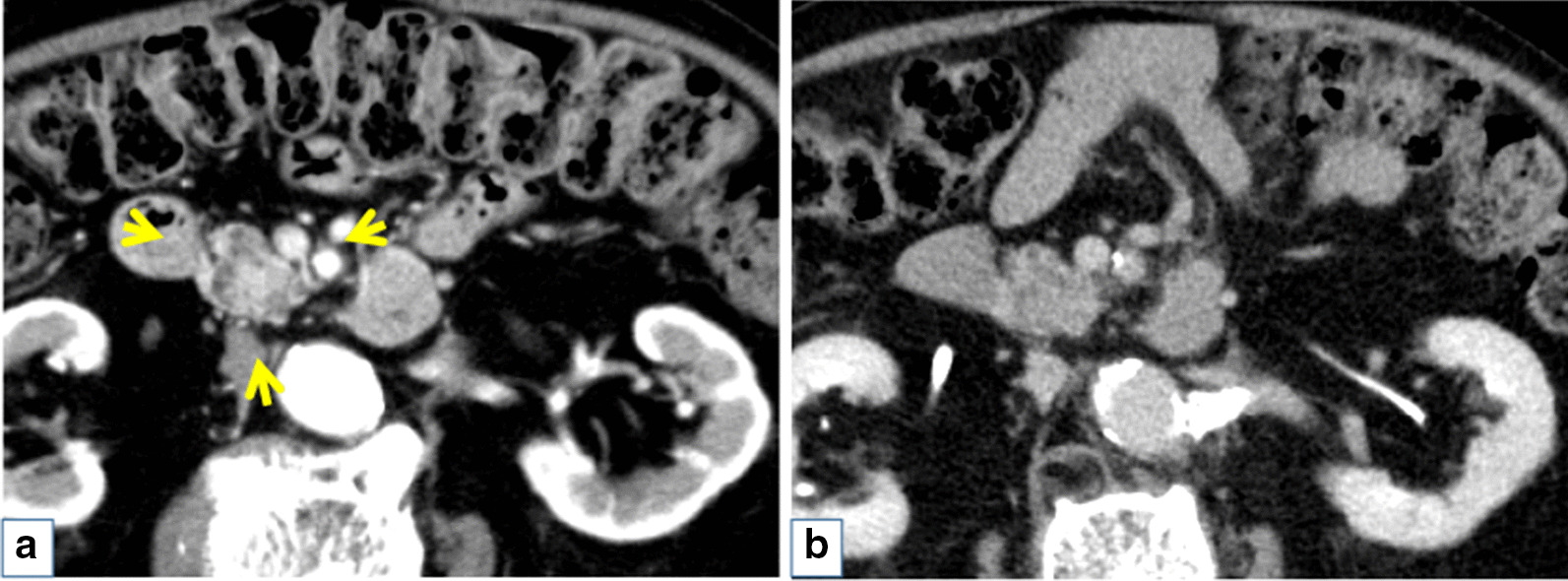
Contrast enhanced computed tomography revealed enhanced round mass sized in 15 mm surrounding with a little fluid (arrow) in early phase (**a**) and the mass was not clearly detected in equilibrium phase (**b**)

**Fig. 2:**
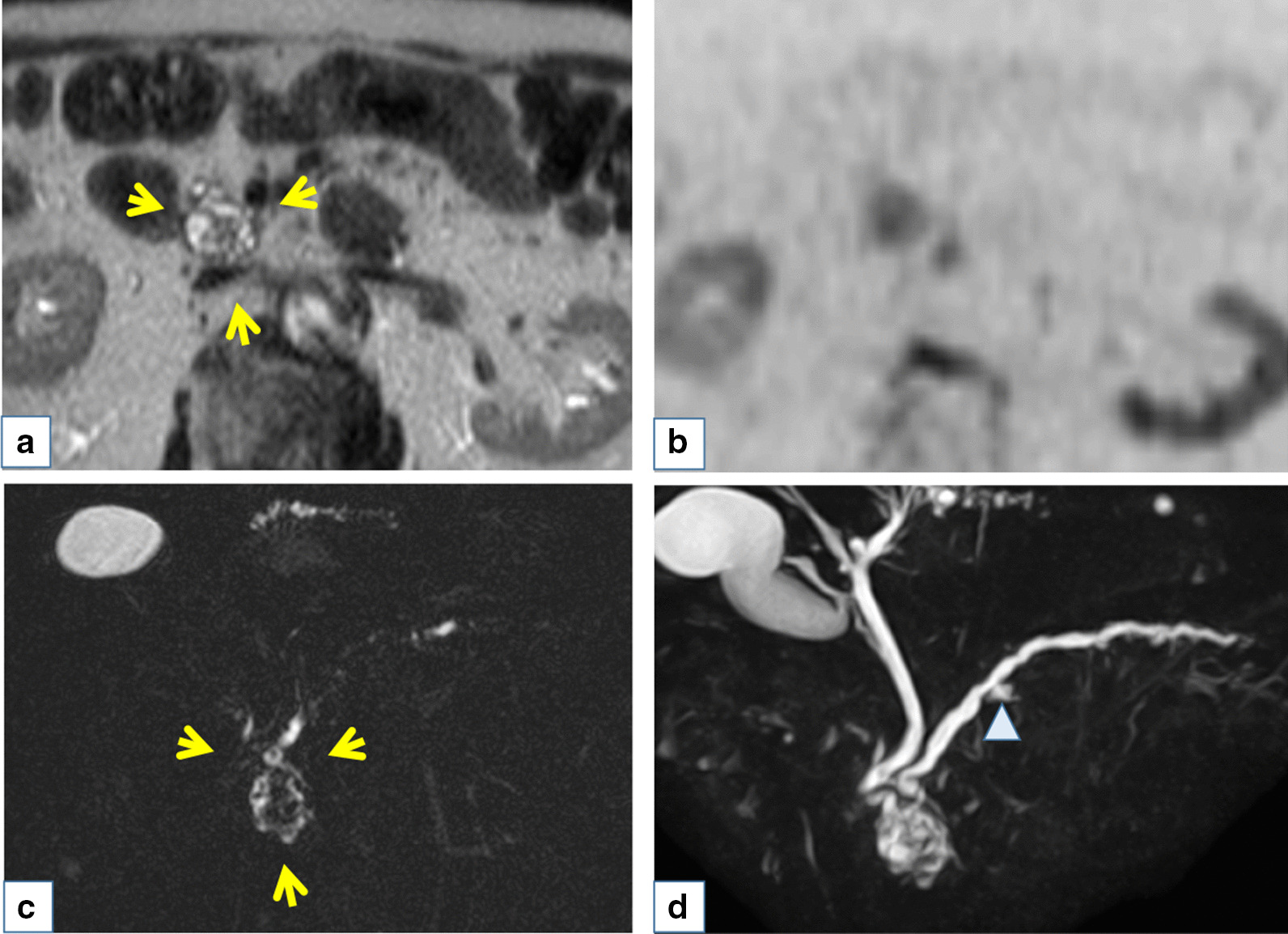
T2 weighted intension picture in magnetic resonance imaging showed 20 mm cystic lesion with internal mass in 15 mm (arrow) (**a**) and the internal mass had high intense in diffusion weighted intention picture (**b**). Magnetic resonance cholangiopancreatography also revealed cystic lesion of the pancreas head with internal mass (arrow) (**c**), slight and diffuse dilatation of main pancreatic duct (MPD), and cyst in 8 mm suspicious for branch duct type intraductal papillary mucinous neoplasmin the pancreatic body (ahead) (**d**)

**Fig. 3:**
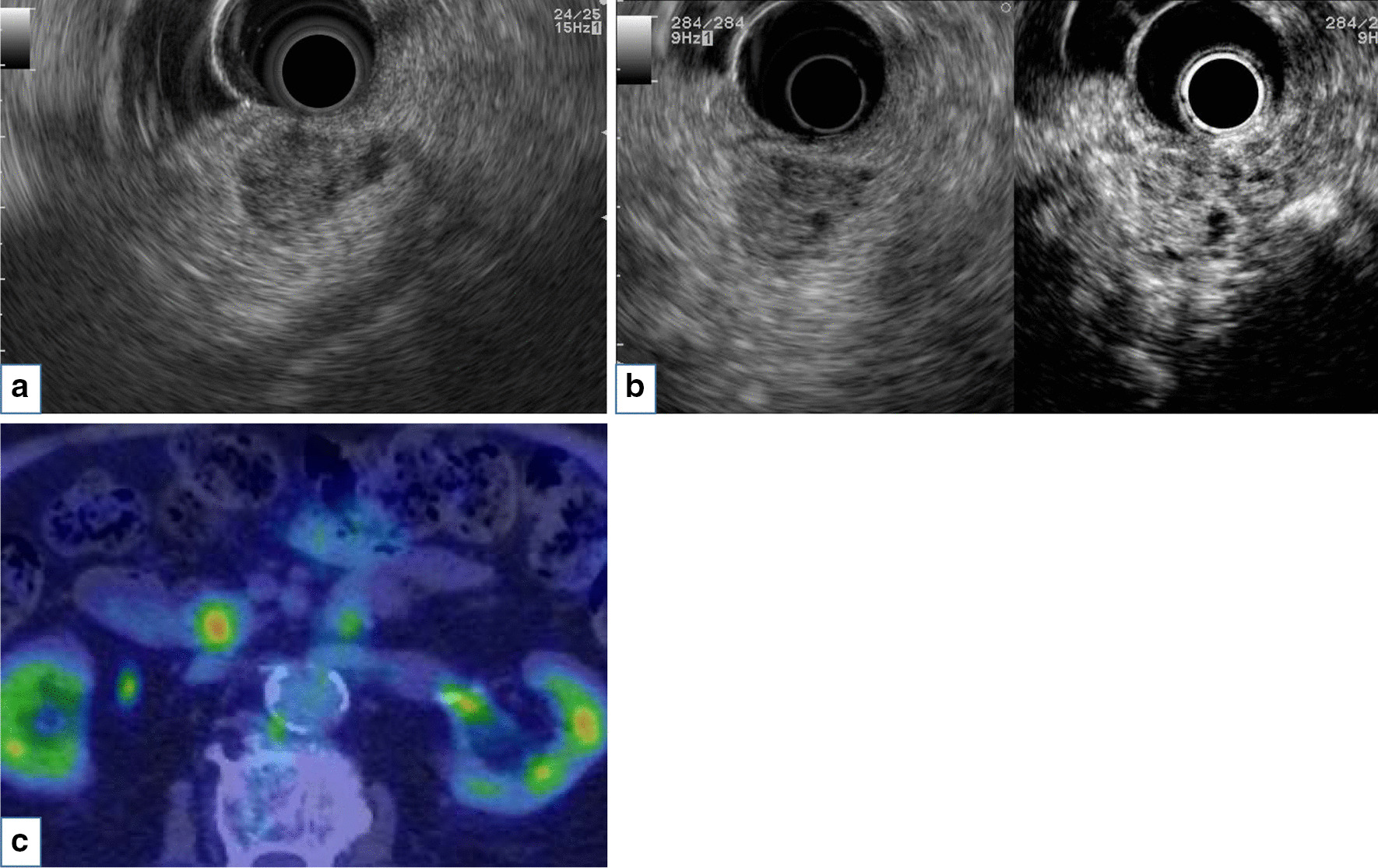
Endoscopic ultrasonography showed 15 mm round hypo echoic mass with fluid collection in its margin (**a**) and contrast enhanced-ultrasonography using Sonazoid showed iso or high vascularity (**b**). Positron emission tomography revealed high accumulation of 2-[^18^F] fluoro-2-deoxy-D-glucose as 4.53 of max standardized uptake value (**c**)

**Fig. 4:**
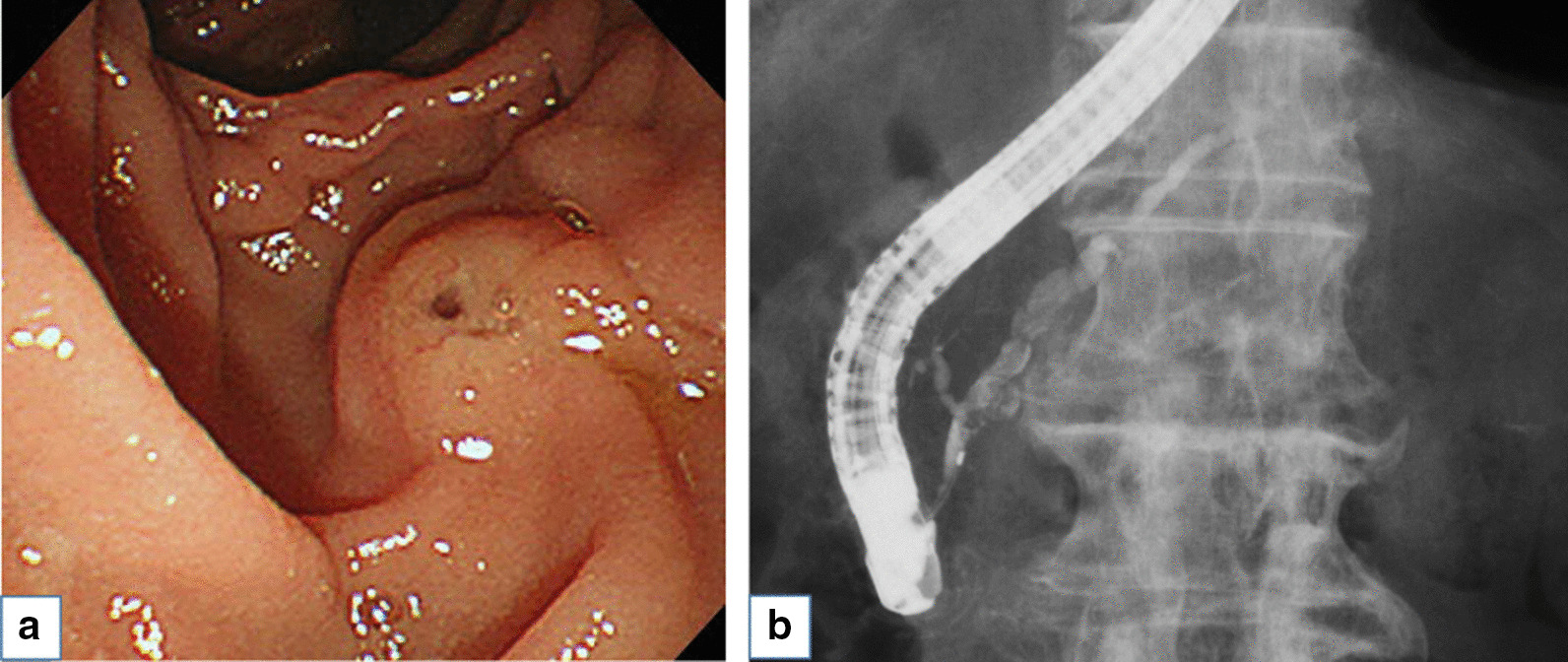
Duodenal papilla was opening slightly (**a**) and Endoscopic retrograde cholangiopancreatography revealed mild and diffuse dilatation of main pancreatic duct (MPD) and contrast defect suspicious of mucus in the MPD (**b**)

The patient was an 85-year-old man who was followed after colonic surgery for rectal carcinoma. Abdominal computed tomography (CT) had been performed once every six months. An abdominal CT scan revealed a cystic mass in the pancreatic head and further examination was done. The patient had hypertension, hyperlipidemia, benign prostate hyperplasia, and a herniated disc in lumbar spine, but no history of chronic pancreatitis. He had surgical resection for rectal cancer at age of 83 years old. The patient did not smoke or drink alcohol. His brother had stomach cancer. Physical examination results on admission were as follows: height, 152 cm, weight, 50 kg, body temperature 36.4℃. His abdomen was soft and flat with no palpable mass. His relevant laboratory data were glutamic oxaloacetic transaminase 34 IU/l, glutamic pyruvic transaminase 45 IU/l, and carcinoembryonic antigen 5.5 ng/ml. Carbohydrate antigen 19–9 and pancreatic enzymes were within a normal range. Contrast enhanced CT revealed an early enhanced round mass sized 15 mm surrounding with a slight amount of fluid (Fig. 1a) and the mass was not clearly detected in the equilibrium phase (Fig. 1b). A T2 weighted intension picture in magnetic resonance imaging showed a 20 mm cystic lesion with an internal mass of 15 mm (Fig. 2a) and the internal mass had a high intensity in the diffusion weighted intention picture (Fig. 2b). Magnetic resonance cholangiopancreatography (MRCP) also revealed a cystic lesion of the pancreas head with an internal mass (Fig. 2c) and slight and diffuse dilatation of MPD and cystic lesion suspicious for branch duct type IPMN in the pancreatic body (Fig. 2d). Endoscopic ultrasonography (EUS) showed a 15 mm round hypo echoic mass with fluid collection in its margin (Fig. 3a) and the mass had iso or high vascularity (Fig. 3b). Positron emission tomography revealed high accumulation of 2-[^18^F] fluoro-2-deoxy-D-glucose as 4.53 of the max standardized uptake value (Fig. 3c). Duodenal papilla were slightly opening and endoscopic retrograde pancreatography (ERP) revealed mild and diffuse dilatation of MPD (Fig. 4a) and mucus in the MPD (Fig. 4b) and examination of the pancreatic juice showed no atypical cells. Fine needle biopsy was not performed because the lesion was intraductal. Considering all the image examinations, we diagnosed the tumor as an IPMN with carcinoma because of its large mural nodule (> 10 mm in size) in the cyst. Although the patient was very high age (85 years old), we performed a pancreatoduodenectomy because of his fair general condition and the possibility of tumor invasion to pancreatic parenchyma.

**Fig. 5:**
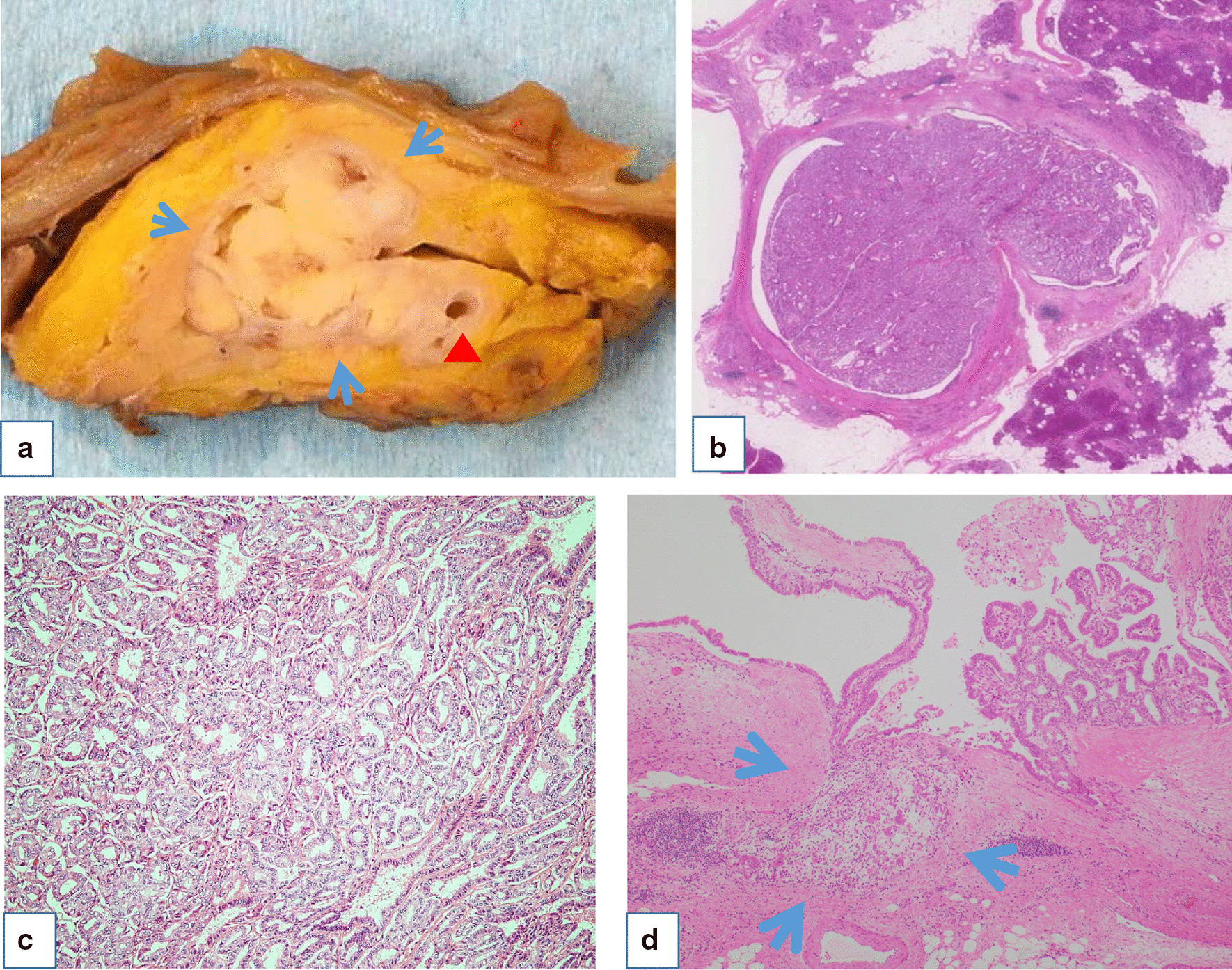
A white solid tumor sized 2.5 × 1.8 × 1.0 was identified in the head of pancreas (arrow) and arrowhead represent the main pancreatic duct (**a**). The cut surface of the resected pancreas showed side-branch type intraductal tumor with tubullopapillary architecture without mucin secretion (**b**). The cells were slight eosinophilic and cuboidal and tumor had grown with tubullary structure in most part (**c**). The tumor had an extraductal invasion of only 1 mm in length (arrows) (**d**)

**Fig. 6:**
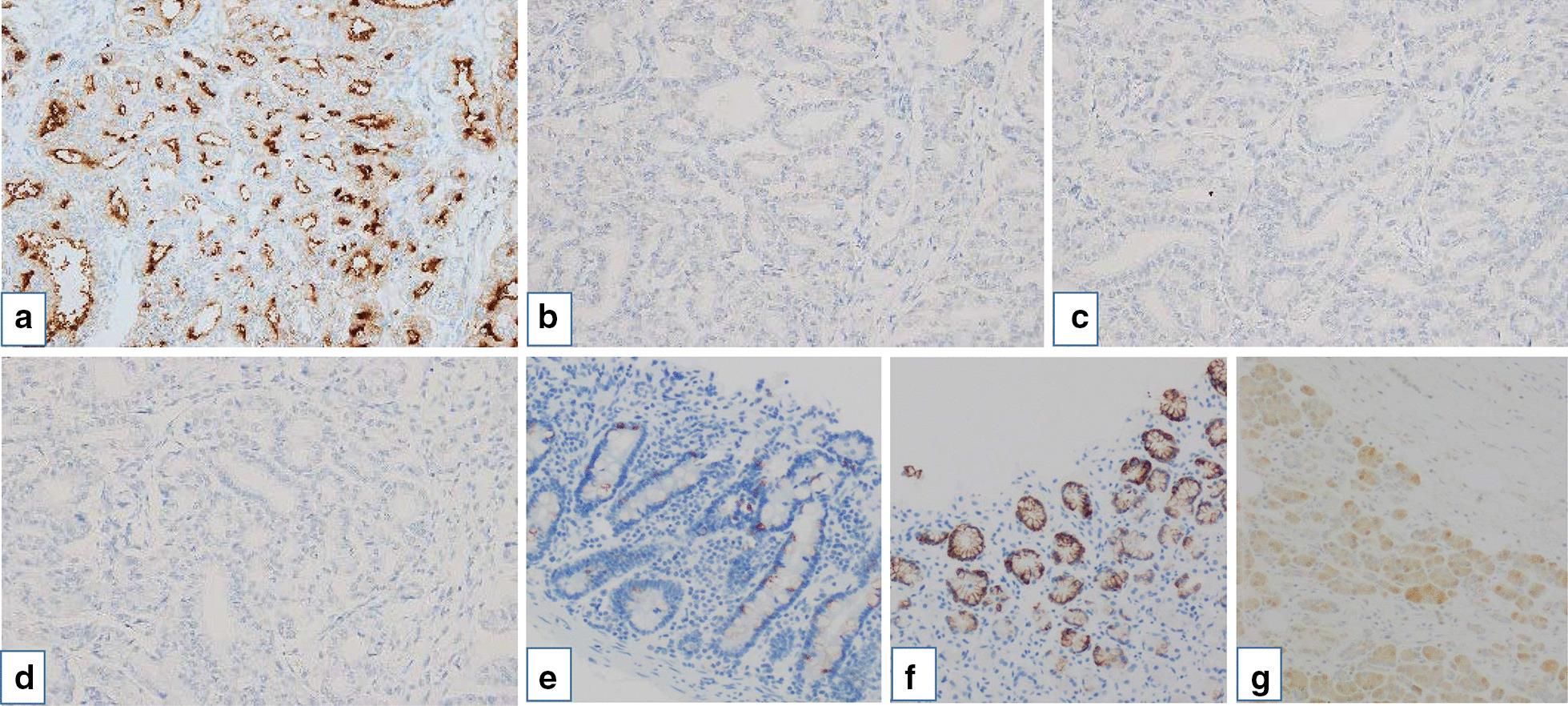
Immunohistochemical staining was positive for MUC1 (**a**), and negative for MUC2 (**b**), MUC5AC (**c**), and trypsin (**d**). Positive control for MUC2 in patient’s intestine (**f**), Positive control for MUC5AC in another patient’s stomach (**g**), Positive control for trypsin in patient’s normal pancreas (**h**)

Macroscopically, a white solid tumor sized 2.5 × 1.8 × 1.0 was identified in the head of the pancreas. The cut surface of the resected pancreas showed a side-branch type intraductal tumor with a tubulopapillary architecture without mucus secretion (Fig. 5a–c). The excision margin was negative. Although the tumor had an extraductal invasion of only 1 mm in length (Fig. 5d), there was no vascular invasion and no lymph node metastasis (0/24). Immunohistochemical staining was positive for MUC1, and negative for MUC2, MUC5AC, MUC6, and trypsin (Fig. 6f). The final diagnosis was determined to be pancreatic ITPN from the branch of the pancreatic duct with minimal invasion to the pancreatic parenchyma. Ki67 index in this case was 3.98%. At the time of this report (48 months post-surgery), the patient remains disease-free without evidence of recurrence. The mucus at ERP was thought to be from the cyst suspicious for branch duct type IPMN in the pancreatic body.

## Discussion and conclusion

ITPN is a new concept regarding pancreatic disease proposed by Yamaguchi et al. in 2009[2] and is classified as an intraductal tumor of the pancreas in the WHO classification in 2010[1]. These tumors grow in the pancreatic duct with a tubulopapillary pattern and are thought to be a disease with borderline malignancy and do not induce a poor prognosis. Yamaguchi et al. stated the following nine characteristics of ITPN: (1) grossly, it is a solid tumor filling the pancreatic duct, (2) there is little mucus, (3) growth is in a tubule-papillary pattern, (4) all epithelium consists of high-grade atypia, (5) there is often a very small necrotic part, (6) immune-histologically, it is positive for CK-7 and CK-9 because of its tubulary differentiation, (7) immune-histologically, it is negative for trypsin because of its lack of acinar differentiation, (8) there is no MUC-2, MUC-5AC, and (9) no mutation in KRAS and BRAF [2]. ITPN is a very rare disease as 3% of intraductal tumors. Its clinicopathological features are not fully understood.

Most cases of ITPNs, previously reported, localized in MPD, not in BD, and the localization is thought to be one of the features of ITPN. We experienced an ITPN localized in BD. This localization is rare and we had difficulty in diagnosis before surgery. Yoshida et al. reported the frequencies of ITPN in BD as 5% (2/41) in 2015 [3]. We collected 49 cases of ITPN from PubMed using the keywords: “intraductal tubulopapillary neoplasm” and “intraductal tubular carcinoma and pancreas,” as well as six cases of ITPN from *Igakutyuozassi* (Japan) using the keywords: “intraductal tubulopapillary neoplasm,” “ITPN,” and “*suikannnai-kannjounyutou-syuyou*” and reviewed these 55 cases and our case.

In 53 cases mentioned about localization, there were 48 cases localized in MPD (35 in MPD and 13 in MPD plus BD) and 5 in BD (9.4%). Five BD cases and a comparison between MPD and BD cases are shown in Tables 1 and 2, respectively. A median tumor size of a BD case was smaller than MPD (BD localized: median 12 mm, MPD: median 32 mm). Moreover, two BD cases were cystic and lacked a solid component. Frequencies of extraductal invasion and lymph node metastasis were higher in MPD cases than BD cases (Table 2), and MPD cases had eight recurrences including 4 distinct metastasis, although BD cases had no recurrence. In addition, the Ki 67 index of tumors was higher in MPD cases than in BD cases (Table 2). These results might show that the malignant potential of ITPN localized in BD is lower than MPD. Past reports have suggested a larger tumor size, male sex, and high Ki67 proliferation associated with risk of invasion [7]. In accordance with these reports, the lower occurrence of invasion and metastasis of BD cases than MPD cases might come from their smaller tumor size, not from their localization in BD. Fortunately, the prognosis of ITPN-associated invasive carcinoma is much better than that of traditional PDAC, even in patients with recurrent and metastatic disease [8]. In this literature review, we found five recurrences in remnant pancreas including three intraductal relapses in MPD cases. Ko et al. [9] discussed the possibility of intraductal colonalization of ITPNs. Although the prognosis of ITPN (in MPD and BD) is thought to be fair, it is important to consider the characteristics of ITPN and intraductal recurrence after surgery.

**Table 1.** 5cases of intraductal tubulopapillary neoplasm developed in branch of the pancreatic duct

Case	Ref.	Age	M/F	Symptom	AP (Y/N)	CT imaging	MPD dilatation	Diagnosis according to imaging	Histological examination	Preoperative diagnosis
1	4	55	F	Upper abdominal pain	N	Multilobular cyst in 50 mm, no nodule	None	BD-IPMN	None	BD-IPMN
2	3	75	M	None	N	Hypovasucular SOL in 12 mm	None	NET, ACC, SCN, PDAC Metastatic tumor	EUS-FNA (s/o ACC)	ACC
3	5	48	M	Abdominal pain	N	Hypovasucular SOL in 20 mm	N.A	SPN, IPMN, NET (cystic)	EUS-FNA: papillary epithelial neoplasm in cyst fluid	Papillary epithelial neoplasm
4	6	41	F	None	N	Multilobular cyst in 25 mm, no nodule	N.A	BD-IPMN	EUS-FNA: marked epitherial atypia in fluid	Marked epitherial atypia
5	Our case	85	M	None	N	Hypervasucular SOL in 20 mm	Slight	BD-IPMN	Cytology using pancreatic fluid in ERP: no malignancy	BD-IPMN

Case	Operation	Localization	Tumor size as nodule (mm)	Invasion	LNM	Distinct metastasis	Immunohistochemical findings					Prognosis
							MUC1	MUC2	MUC5AC	MUC6	Ki67 (%)	
1	PD	Head	No nodule	None	None	None	●	×	×	●	1	Survival with no recurrence for 34 month
2	DP	Head	12	None	None	None	●	N.A	×	●	N.A	N.A
3	DP	Body–tail	13	None	None	None	●	N.A	×	N.A	N.A	N.A
4	PD	Head	No nodule	None	None	None	●	×	N.A	N.A	5	N.A
5	PD	Head	24	Minimally stromal invasion	None	None	●	×	×	×	3.98	Survival with no recurrence for 48 month

Ref. = reference, M = male, F = female, AP = acute pancreatitis, Y = yes, N = none, SOL = space occupied lesion, N.A = not available, MPD = main pancreatic duct, BD-IPMN = branch duct type intraductal papillary mucinous neoplasm, NET = neuroendocrine tumor, ACC = acinar cell carcinoma, SCN, serous cystic neoplasm, PDAC = pancreatic ductal adenocarcinoma, EUS-FNA = fine needle aspiration using endoscopic ultrasonography, ERP = endoscopic retrograde pancreatography

PD = pancreatoduodenectomy, DP = distal pancreatectomy, LNM = lymph node metastasis, ● = positive, ×  = negative, N.A = not available

**Table 2:** Comparison of features between ITPN in MPD and BD

		MPD (N = 48)^a^	BD (N = 5)^a^	P value^c^
Gender	M:F	30:18	3:2	0.63
Age	Mean ± SD	63 ± 13	61 ± 19	0.74
Tumor size^b^ (solid part)	Median (range)	32 (10–150)	12 (0–24)	< 0.01
Invasion	Yes (%)	26 (52%)	1 (20%)	0.13
	Minimally invasive–intra-pancreas	15	1	
	Extra-pancreas–infiltration of other organs	8		
	Unknown	3		
	None	19	4	
	N.A	3		
Lymph node metastasis	Yes (%)	3 (8.8%)	0	0.66
	None	32	5	
	N.A	13	0	
Distant metastasis	Yes	0	0	
	None	35	5	
	N.A	13	0	
Ki 67 (%)	Median (range) (N)	24.6 (1–65) (N = 29)	3.98 (1–5) (N = 3)	0.33
Relapse		7	0	0.48
	Pancreatic duct	3		
	Pancreatic parenchyma	2		
	Liver metastasis	1		
	Rectal metastasis	1		
Death from the ITPN		0	0	

ITPN = intraductal tubulopapillary neoplasm of the pancreas, M = male, F = female, N.A = not available, MPD = the main pancreatic duct, BD = branch of the pancreatic duct

^a^The localization of tumor, whether in MPD or BD, were not available in 3 cases out of 56 cases

^b^Tumor size in case of growing over the entire pancreas was treated to be 100 mm

^c^For statistical analysis, Fisher’s exact test was used for categorical variables and the Welch *t*-test and Mann–Whitney *U* test were for quantitative data

Diagnosis of ITPN is difficult if localized in a branch duct. First, there are few symptoms compared to ITPNs in MPD. ITPNs in MPD often induce more symptoms and pancreatitis than ITPNs in BD (symptom 56.7% (29/51) in MPD, 40% (2/5) in BD, acute pancreatitis (23.8% (10/42) in MPD, none in BD) (Table 2).

Furthermore, the preoperative pathological diagnosis of ITPN in BD is very difficult. There is a possibility in preoperative histological diagnosis of ITPN in MPD with fine needle aspiration using EUS (EUS-FNA) or biopsy or cytology via the pancreatic duct. Although, a biopsy via the pancreatic duct of ITPN in BD is difficult. Regarding to EUS-FNA, firstly, EUS-FNA for cystic neoplasm is still tends to be avoided in Japan because of worrisome for peritoneal dissemination. Second, there were no ITPN in BD diagnosed before surgery in literature review. In three of five BD cases, histocytological examination with EUS-FNA was performed, but a final diagnosis of ITPN was not obtained. Two cases had a diagnosis of epithelial neoplasm [5, 6]. Yoshida et al. [3] arrived at a diagnoses of suspicion of acinar cell carcinoma without immunohistochemical examination and stated the need for imunohistochemical examination. Currently, there have been few cases localized in BD, thus additional BD cases are needed to understand how to diagnose ITPN in BD before surgery.

In accordance with the difficulty of obtaining a histological diagnosis, we need to diagnose using imaging examinations. The features of ITPN in MPD are characterized as a hypo dense solid mass and dilatation of upstream MPD, which is similar with pancreatic ductal adenocarcinoma. A study by Motosugi [10] et al. described a 2-tone duct sign and a cork-of-bottle sign as helpful imaging findings of ITPN. The 2-tone duct signs were composed of a higher density tumor area, occluding the main pancreatic duct, and a lower density luminal area dilatated in the upstream lumen and the cork-of-wine-bottle sign is when the tumor is surrounded by pancreatic fluid in the dilated duct. Both of these images are obtained in patients with ITPN in MPD.

ITPN in BD have only a small amount of marginal fluid, so we need to distinguish serous cystic neoplasm, degeneration of tumor (ex; neuroendocrine neoplasm, acinar cell carcinoma (ACC) and solid pseudo-papillary neoplasm). For these reasons, it is first necessary to find the mass as an intraductal lesion. At this time, it is possible to recognize the tumor as an intraductal mass using MRCP and contrast enhanced EUS. When we recognized the tumor as an intraductal lesion, we needed to nominate branch duct type IPMN and ACC as a differential diagnosis. Most likely, it is not possible to distinguish the two cystic BD cases without a nodule from the branch duct type IPMN unless there is critical information from EUS-FNA. The histologic images of three BD cases were very similar. The tumors were round in shape, not papillary, and had little fluid. This is likely due to little papillary growth and a lack of fluid including mucus. These characteristics are different from branch type IPMN and can be clues to differentiate ITPN in BD from branch duct type IPMN. Differentiation of ITPN from ACC growing in pancreatic duct is still difficult thru imaging examinations [11].

In conclusion, we experienced a case of ITPN in BD. These tumors are presumed to have a fair prognosis, but diagnosis pre-surgery is very difficult. The round shape of the margin and little surrounding fluid can be characteristics of ITPN in BD. Additional cases of ITPN in BD are necessary for analysis and to support our current findings.

## Data Availability

Not applicable.

## Abbreviations

ITPN: Intraductal tubulopapillary neoplasm
IPMN: Intraductal papillary mucinous neoplasm
MPD: Main pancreatic duct
PDAC: Pancreatic ductal adenocarcinoma
BD: Branch of pancreatic duct
CT: Computed tomography
MRCP: Magnetic resonance cholangiopancreatography
EUS: Endoscopic ultrasonography
ERP: Endoscopic retrograde pancreatography
EUS-FNA: Fine needle aspiration using EUS
ACC: Acinar cell carcinoma
